# Physical activity in children with Juvenile Idiopathic Arthritis compared to controls

**DOI:** 10.1186/s12969-016-0102-8

**Published:** 2016-07-07

**Authors:** G. J. F. Joyce Bos, Otto T. H. M. Lelieveld, Wineke Armbrust, Pieter J. J. Sauer, Jan H. B. Geertzen, Pieter U. Dijkstra

**Affiliations:** Department of Rehabilitation Medicine, University of Groningen, University Medical Center Groningen, PO Box 30001, 9700 RB Groningen, The Netherlands; Department of Pediatric Rheumatology, University of Groningen, University Medical Center Groningen, Beatrix Children’s Hospital, PO Box 30001, 9700 RB Groningen, The Netherlands; Department of Oral and Maxillofacial Surgery, University of Groningen, University Medical Center Groningen, PO Box 30001, 9700 RB Groningen, The Netherlands; University of Groningen, University Medical Center Groningen, Beatrix Children’s Hospital, PO Box 30001, 9700 RB Groningen, The Netherlands

**Keywords:** Physical activity, Juvenile idiopathic arthritis, Physical activity level

## Abstract

**Background:**

To compare physical activity (PA) in children with juvenile idiopathic arthritis (JIA) with controls and to analyse the effect of disease specific factors on PA in children with JIA treated according to current treatment regimes.

**Methods:**

PA was measured with a 7-day activity diary and expressed as physical activity level (PAL). Moderate to vigorous physical activity (MVPA) (hours/day) and sedentary time (hours/day) was determined. In children with JIA, medication, the number of swollen and/or painful joints, disease activity, functional ability, pain and well-being was determined. Multivariate regression analysis was performed to analyze differences in PA between JIA and controls, adjusted for influences of age, gender, season, Body Mass Index (BMI) and to analyze predictors of PA in JIA patients.

**Results:**

Seventy-six children with JIA (26 boys and 50 girls, mean ± SD age 10.0 ± 1.4 years) and 131 controls (49 boys and 82 girls, mean ± SD age 10.4 ± 1.2 years) participated in this study. Children with JIA had a significantly lower PAL (0.10, *p* = 0.01) corrected for age, BMI, gender and season. They spent less time in MVPA (0.41 h/day, *p* = 0.06) and had a significantly higher mean time spent in sedentary activities (0.59 h/day, p 0.02) compared to controls. The activity level of children with JIA was related to age, gender, season, feeling of well-being and pain.

**Conclusion:**

Children with JIA have a lower PAL, spent less time in MVPA and spent more time on sedentary activities compared to controls despite current medical treatment and PA encouragement.

**Trial registration:**

Data of the children with JIA are from the Rheumates@work study ISRCTN92733069.

## Background

The treatment of Juvenile Idiopathic Arthritis (JIA) has changed in the past decade, due to insights in pathogenesis and the availability of new medication biologic drugs [1]. The present aim of treatment is to achieve remission within 3 to 6 months [2] and therefore it is current practice in our institutions to administer a top down medication regime. It is expected that the new treatment options reduce the burden of having JIA including improved physical activity (PA) levels. Studies conducted a number of years ago showed a lower level of PA in children with JIA than controls [3, 4]. A low level of PA in healthy individuals is related to a higher incidence of overweight and hypertension in later life. This low level of PA might even be more dangerous for children with JIA, as they also have signs of inflammation, perhaps increasing the risk of cardiovascular diseases in later life [5–7].

In children with JIA, it was previously assumed that PA could damage joints and as a consequence rest was often prescribed especially when there were indications of disease activity. More recently, activity is more encouraged in children with JIA and PA is considered to be safe [8–10]. In the Netherlands, there is consensus to encourage children with JIA to be physically active even when there are signs of active disease. However, some care providers remain concerned about the level of PA and competitive sports are often not recommended when there is damage or inflammation of the joints even though exercise does not exacerbate arthritis [11].

It is unknown if the treatment advances in children with JIA and the encouragement of PA has led to PA in children with JIA similar to that of healthy controls. The aim of this study was to compare PA in children with JIA who have been treated according to the latest guidelines [12] to controls and to analyse the effect of disease specific factors on PA in children with JIA.

## Methods

### Patients

This study is part of a larger study to measure and promote PA in children with JIA. In total 308 children, aged 8 up to 13, from the Beatrix Children’s Hospital of the University Medical Center Groningen, the Wilhelmina Children’s Hospital of the University Medical Center Utrecht and Amsterdam Rehabilitation Center Reade, all in the Netherlands, were asked to participate in the Rheumates@work study (ISRCTN92733069). Rheumates@work is an internet-based cognitive behavioral intervention to promote PA in children with JIA [13, 14]. All subtypes of JIA, according to the International League Association of Rheumatology, were eligible [15]. Other inclusion criteria beside age and JIA diagnosis were good comprehension of the Dutch language and the availability of a computer with internet connection. Exclusion criteria were high disease activity, defined as visual analogue scale (VAS) as assessed by the pediatric rheumatologist of more than 2 (on a scale of 0 to 10), receiving cognitive behavioural therapy, or patients with physical disability caused by secondary chronic conditions that limited the patients motor and or exercise performance. Children were recruited by the pediatric rheumatologist and received a patient information letter between January 2011 and September 2012. Data of children with JIA were collected twice a year (January and September). Therefore January was labeled as ‘winter’ and data collected in September as ‘summer’.

Eighty-two (27 %) children agreed to participate and parents signed informed consent.

Reference data were collected in the summer of 2009 from a control group of 131 children, age 8 to 13 years, without a mental or physical disability. All children attended one of the last four grades of two Dutch primary schools. One school was located in the countryside and the other in the city. Healthy children were recruited by physiotherapy students. Children and parents received an information letter and a folder. Informed consent was given by the parents.

### Disease activity

Disease activity was assessed according to the core set established by the American College of Rheumatology [16]. Laboratory measures of inflammation were not determined. JIA patients were assessed by a pediatric rheumatologist. Joints were counted as having active disease when they were swollen and/or painful. The pediatric rheumatologist gave a total assessment of disease activity on a VAS, range 1 to 10 centimeter (a higher score corresponded with more disease activity).

Data collection of this study is from the Rheumates@work study in which we have chosen to use VAS to assess disease activity in order to have a measurement of disease activity in major joints instead of the overall measure of the Juvenile arthritis disease activity score (JADAS). The VAS was used to separate children who might be able to increase PA from those who might not be able to do so.

In our study we were also interested in how major joint activity might have an effect on PA and therefore also used VAS as a measure of disease activity in our analysis.

### Functional ability

To assess functional ability, the Childhood Health Assessment Questionnaire (CHAQ-38) was used [17], a revised version of the CHAQ-30 with 8 additional items [18, 19]. It assesses 9 domains: dressing, grooming, arising, eating, walking, hygiene, reach grip, activities and extra-curriculum activities. The scores are converted to a CHAQ disability score with a range between 0 to 3 (a higher score corresponds to more disability). The CHAQ-38 includes a VAS (0–10 cm) for assessment of pain and a VAS (0–10 cm) for evaluation of well-being (a higher score corresponds to more pain and worse overall well-being). The VAS score of pain and well-being were scored by the children themselves.

### Activity diary

The diary of Bouchard was used to record the level of daily PA [20]. Children and parents received an oral and written explanation how to fill in the diary for 7 consecutive usual days during a school week and weekend. Activities are divided into 9 categories according to their average energy cost, 1 representing the lowest activity category (lying, sleep or rest in bed) and 9 representing the highest activity category (competitive sports). For each 15 min the dominant activity was scored. A total of 96 data points were collected per day in the activity diary that was given to the children on paper; for each day one paper bound together with the instructions on top. The children and parents were instructed to fill in the diary during the day period, in case it was not possible to do so, they had to fill in the diary whenever they had the opportunity, but at least once every day. Parents received instructions also on how to support their children in filling in the diary. If the number for the activity was unclear, the instruction given was to describe the activity so the investigators could assign the correct category for the activity. In case of missing data, children were contacted and asked to fill in the missing data. If children could not recall the activity, missing data from 9 pm until 7 am were imputed as a sleeping activity (code 1). Some children filled in 2 values for the same 15 min period. In that case, the first and second values were chosen alternately throughout the diary. In case of less than 4 missing values, the missing values were imputed by a 2 (sitting activities). If more than 4 values were missing in the diary for one day, that day was excluded for further analysis. In case the same weekday was recorded twice in one diary (for instance 2 Mondays), one day was excluded and totals were divided over 6 instead of 7 days. An activity diary had to include at least 3 weekdays and 1 weekend day to be used in this study. Lying and sitting (code 1 and 2) were considered as sedentary activities. Light PA was defined as codes 3–5, moderate to vigorous PA (MVPA) by codes 6–9.

PA in this study was defined as Physical activity level (PAL), MVPA and as sedentary time. PAL is an average value, which includes the energy cost of all activities over a 24-h period [21]. PAL is calculated by dividing total energy expenditure by Basic Metabolic Rate (Appendix 1) [22]. The basis of PAL was formulated in the FAO/WHO/UNU expert committee on energy requirements [21]. Mean time spent in MVPA (hours/day) and mean sedentary time (hours/day) was calculated over 7 days. The number of days obtaining the PA guidelines of at least 1 h of MVPA each day were counted.

### Statistical analysis

For the statistical analysis IBM SPSS statistics version 22 was used. The effect of the season on PA in children with JIA was analyzed using an independent samples *t*-test. Multivariate regression analysis (method enter) was performed to analyze differences in PA between JIA and controls, adjusted for influences of age, gender, seasonal influence, and Body Mass Index (BMI) and to analyze predictors of PA in children with JIA. Potential predictors of PA in children with JIA were BMI, gender, age, season, functional ability, medication and global assessment of disease activity. The pediatric rheumatologist assessed the global assessment of disease activity and each child pain and overall well-being. Data about BMI and age were centered by their means. Results while on and off medication were entered in the regression model. A *p*-value of 0.05 or less was considered significant. In the regression analyses, interaction effects were explored if main effects were significant. Residuals were checked for a normal distribution.

## Results

A total of 82 children with JIA and 131 controls filled in the activity diary. Data of 6 children with JIA were excluded from the analysis because of missing data. Seven diaries of children with JIA and 2 diaries of controls included data for 6 days. One diary of a child with JIA included 5 days (Table 1).

**Table 1:** Characteristics of children with juvenile idiopathic arthritis and controls

	*JIA (n = 76)*	*Controls (n = 131)*	*95 % CI lower*	*95 % CI upper*	*P*
Gender, boys (%)	26 (34 %)	49 (37 %)			
Age, years	10.0 ± 1.4	10.4 ± 1.2	−0.75	−0.02	0.04
Weight, kg	35.6 ± 9.0	38.5 ± 9.1	−5.47	−0.34	0.03
Height, cm	143.3 ± 10.1	148.5 ± 9.7	−7.93	−2.31	<0.01
BMI, kg/m^2^	17.1 ± 2.9	17.3 ± 2.6	−0.89	0.64	0.75
Physical activity					
• Physical activity level (per day)	1.6 ± 0.2	1.8 ± 0.2	−0.25	−0.14	<0.01
• Time spent in MVPA (hours/day)	1.3 ± 0.8	2.1 ± 1.2	−1.02	−0.47	<0.01
• Sedentary time (hours/day)	19.3 ± 1.3	18.2 ± 1.3	0.69	1.43	<0.01
Total days per week meeting public health recommendations	3.9 ± 1.7	4.9 ± 1.6	−1.45	−0.54	<0.01
Time since diagnosis (years)	3.6 ± 2.7				
Disease activity					
VAS physicians global assessment (cm)	0.3 (0–0.9)				
Number of active joints	1.0 (0–1.0)				
Upper extremity	0 (0–0)				
Lower extremity	1.0 (0–1.0)				
Number of limited joints	1.0 (0–2.0)				
Functional ability (CHAQ)	0.3 (0.1–0.8)				
VAS pain (cm)	1.5 (0.2–3.9)				
VAS well-being (cm)	0.8 (0.2–2.6)				

Values are the mean ± standard deviation. For disease activity, number of limited joints, functional ability, VAS pain and VAS well-being values are in median (25^th^ and 75^th^ percentiles). Number of valid observations for age in controls *n* = 127, height and BMI in controls *n* = 129

*JIA* juvenile idiopathic arthritis, *CI* confidence interval, *MVPA* moderate to vigorous physical activity, *BMI* body mass index, *CHAQ* childhood health assessment questionnaire, *VAS* visual analogue scale, *cm* centimeter, *kg* kilogram, *m* meter Number of days per week meeting public health recommendations were counted per day of which at least 1 h of MVPA was present

Of the 76 children with JIA included, 9 % (7) had systemic JIA, 33 % (25) had persistent oligoarticular JIA, 13 % (10) extended oligoarticular JIA, 36 % (27) were classified as having polyarticular JIA (of which 11 % (3) with a positive rheumatoid factor), 5 % (4) had psoriasis related JIA and 4 % (3) had enthesitis related JIA.

Of the children with JIA 75 % (57) were on medication, 36 % (27) did not have any disease activity according to the assessment by the pediatric rheumatologist and 46 % (35) of the children with JIA did not have any swollen and/or painful joints.

Children with JIA had a lower PAL, spend less time in MVPA and spend more time on sedentary activities as shown in Table 1. In children with JIA, 4 % (3) met the PA recommendations of spending at least 1 h a day in MVPA. In controls 16 % (21) achieved that standard (Table 1). On average, children with JIA had close to 4 days of meeting this PA recommendation compared to 5 days a week in controls.

Data of children with JIA was collected twice a year. A difference in data collected in the summer and winter was found. The children whose data was collected in the summer had a significantly higher PAL and spent significantly less time in sedentary activities compared to the winter. No difference in seasonality was found in time spent in MVPA (Table 2). Seasonality was entered in the regression analyses. Residuals of the regression analyses were normally distributed. The multivariate linear regression analysis, when corrected for the effects of age, BMI, gender and season, showed that children with JIA have a significantly lower PAL (0.10, *p* = 0.01), spend significantly more time on sedentary activities (0.59 h/day, *p* = 0.02) and less time in MVPA (0.41 h/day, *p* = 0.06) (Table 3).

**Table 2:** Seasonal influence on physical activity in children with juvenile idiopathic arthritis

	*Summer n = 34*	*Winter n = 42*	*95 % CI lower*	*95 % CI upper*	*P*
Physical activity					
• Physical activity level (per day)	1.7 ± 0.1	1.6 ± 0.2	0.01	0.17	0.03
• Time spent in MVPA (hours/day)	1.5 ± 0.7	1.2 ± 0.9	−0.12	0.63	0.18
• Sedentary time (hours/day)	18.9 ± 1.2	19.6 ± 1.4	−1.33	−0.13	0.02
Total days per week meeting public health recommendations	4.2 ± 1.6	3.7 ± 1.7	−0.28	1.27	0.21

Values are the mean ± standard deviation

*CI* confidence interval, *MVPA* moderate to vigorous physical activity

**Table 3:** Multivariate linear regression analyses to predict physical activity in children with juvenile idiopathic arthritis and controls

	*B*	*95 % CI lower*	*95 % CI upper*	*P*
PAL				
Reference	1.75	1.67	1.83	<0.01
Controls	0.10	0.03	0.18	0.01
Age centered 10 years	0.04	0.02	0.07	<0.01
BMI centered 17 kg/m2	−0.01	−0.02	−0.00	0.04
Gender	−0.07	−0.13	−0.01	0.02
JIA season	−0.14	−0.23	−0.05	<0.01
MVPA				
Reference	1.70	1.27	2.13	<0.01
Controls	0.41	−0.02	0.83	0.06
Age centered 10 years	0.20	0.07	0.33	<0.01
BMI centered 17 kg/m2	−0.02	−0.08	0.04	0.50
Gender	−0.12	−0.43	0.20	0.47
JIA season	−0.50	−1.00	0.01	0.06
Sedentary time				
Reference	18.86	18.36	19.37	<0.01
Controls	−0.59	−1.09	−0.09	0.02
Age centered 10 years	−0.13	−0.28	0.03	0.10
BMI centered 17 kg/m2	0.15	0.08	0.21	<0.01
Gender	−0.02	−0.38	0.35	0.92
JIA season	0.78	0.18	1.37	0.01

The regression equation for PAL is as follows:

$$ \begin{array}{l}\mathrm{PAL}\kern0.5em =\kern0.5em \mathrm{reference} + 0.10\ *\ \mathrm{control} + 0.04\ *\mathrm{age}\ \left(\mathrm{centered}\ 10\right) + -0.01\ *\ \mathrm{B}\mathrm{M}\mathrm{I}\ \left(\mathrm{centered}\ 17\right) + -0.07\ \\ {}*\ \mathrm{gender} + -0.14\ *\ \mathrm{season}\end{array} $$

The reference for this equation is a 10 year old boy with JIA, a BMI of 17 kg/m^2^ of which the data was collected in the summer. So a healthy girl (no JIA) of 8 years old, a BMI of 20 has a predicted PAL of (1.75 + 0,10 * 1 + 0.04 * (8–10) + −0.01 * (20–17) + −0.07 * 1 = 1.73

*JIA* juvenile idiopathic arthritis, *BMI* body mass index, *PAL* physical activity level, *MVPA* moderate to vigorous activity expressed in hours/day. Sedentary time expressed in hours/day, *CI* confidence interval of B. Reference category: Boy of 10 years, with a BMI of 17, with JIA, who filled in the diary in the summer period

In Table 4, the results are given of the predicted PA in children with JIA. A lower PAL in children with JIA was associated with young age, seasonality (winter) and worse well- being and less pain. The same associations were found for time spend in MVPA and sedentary time. We found no association between disease activity as accessed by the pediatric rheumatologist as well as use of medication (on/off) with PA in children with JIA. In mean time spend in MPVA, we also found an association with functional ability (CHAQ). A higher CHAQ score was associated with less time spend in MVPA. For sedentary time an association was found in BMI; a higher BMI corresponds with more time spend in sedentary activities. No significant interaction effects were found.

**Table 4:** Multivariate linear regression analyses to predict physical activity in children with juvenile idiopathic arthritis

	*B*	*95 % CI lower*	*95 % CI upper*	*P*
PAL				
Reverence	1.81	1.72	1.90	<0.01
Age centered 10 years	0.06	0.03	0.09	<0.01
BMI centered 17 kg/m^2^	−0.01	−0.02	0.00	0.18
Gender	−0.07	−0.14	0.01	0.08
JIA season	−0.16	−0.23	−0.08	<0.01
Medication	−0.01	−0.09	0.07	0.87
Disease activity	−0.005	−0.012	0.003	0.83
Functional ability (CHAQ)	−0.05	−0.15	0.04	0.27
VAS well-being	−0.04	−0.07	−0.01	0.01
VAS pain	0.03	0.002	0.05	0.04
MVPA				
Reference	2.00	1.52	2.48	<0.01
Age centered 10 years	0.26	0.10	0.41	<0.01
BMI centered 17 kg/m^2^	0.001	−0.07	0.07	0.99
Gender	−0.13	−0.51	0.25	0.51
JIA season	−0.55	−0.96	−0.15	0.01
Medication	0.03	−0.39	0.44	0.90
Disease activity	−0.01	−0.03	0.02	0.60
Functional ability (CHAQ)	−0.50	−0.99	0.01	0.05
VAS well-being	−0.16	−0.30	−0.02	0.03
VAS pain	0.13	0.01	0.26	0.03
Sedentary time				
Reference	18.70	17.94	19.46	<0.01
Age centered 10 years	−0.28	−0.53	−0.04	0.02
BMI centered 17 kg/m^2^	0.16	0.05	0.27	<0.01
Gender	0.07	−0.54	0.67	0.83
JIA season	1.01	0.38	1.67	<0.01
Medication	−0.29	−0.96	0.37	0.39
Disease activity	0.01	−0.03	0.05	0.69
Functional ability (CHAQ)	0.14	−0.65	0.94	0.72
VAS well-being	0.26	0.04	0.48	0.02
VAS pain	−0.19	−0.38	0.00	0.05

The regression equation for PAL is as follows:

$$ \begin{array}{l}\mathrm{PAL} = \mathrm{reference} + 0.06\ *\ \mathrm{age}\ \left(\mathrm{centered}\ 10\right) + \hbox{-} 0.01\ *\ \mathrm{B}\mathrm{M}\mathrm{I}\ \left(\mathrm{centered}\ 17\right) + \hbox{-} 0.07\ *\ \mathrm{gender} + \hbox{-} 0.16\ \\ {}*\ \mathrm{season} + \hbox{-} 0.01\ *\ \mathrm{medication} + \hbox{-} 0.005\ *\ \mathrm{disease}\ \mathrm{activity} + \hbox{-} 0.05\ *\ \mathrm{functional}\ \mathrm{ability}\ \left(\mathrm{CHAQ}\right) + \hbox{-} 0.04\ \mathrm{V}\mathrm{A}\mathrm{S}\ \\ {}\mathrm{well}\hbox{-} \mathrm{being} + 0.03\ *\ \mathrm{V}\mathrm{A}\mathrm{S}\ \mathrm{pain}. = \end{array} $$

The reference in this equation a 10 year old boy with JIA, a BMI of 17 kg/m^2^ of which the data was collected in the summer and off medication

*JIA* juvenile idiopathic arthritis, *BMI* body mass index, *PAL* physical activity level. MVPA: moderate to vigorous activity expressed in hours/day. Sedentary time expressed in hours/day. *CI* confidence interval of B. Reference category: Boy of 10 years, with a BMI of 17, with JIA, who filled in the diary in the summer period and is off medication. A lower score in well-being corresponds to a better well-being. CHAQ, childhood health assessment questionnaire

## Discussion

This study shows that the physical activity level of children with JIA, treated according to recent treatment guidelines [12], is lower compared to controls. PA was not associated with medication or disease activity as measured by the pediatric rheumatologist, but with patient’s assessment of well-being and pain score.

Previous studies also found that children with JIA are less active compared to controls [3, 4, 23–25]. The lower level of PA seems to persist in these children, despite education regarding the importance of an active lifestyle, as well as when some signs of disease activity are present.

In this study we found that only a minority of children with JIA (4 %) and healthy children (16 %) met the recommendations for normal PA (e.g. spending at least 1 h in MVPA each day of the week) [26]. Other studies reported that 38 % of children with JIA and 60 % of controls [23] and 23 % of adolescents with JIA and 66 % of controls [4] did meet PA recommendations [26]. In a report of Dutch children on PA and health, a trend of decline in meeting the recommendations for normal PA over the years is seen in the period of 2006–2014 in the ages of 4 to 17 year. No specific reason is given as to why this decline occurred [27]. It is alarmingly that this level of PA is declining, especially since the levels of PA in children with JIA are even lower compared to controls. For health benefits, it is desirable that more children meet these PA recommendations. Additionally, both children with JIA and controls spend much time in sedentary activities. More time spent sitting during the day is associated with increased risks of mortality and cardiovascular disease and all causes. Even when individuals are very active, an association between sitting time and mortality has still been found [28].

Contrary to our expectations, we found a positive association between pain as indicated by the children with JIA and the level of activity, that PAL and time spent in MVPA increased with more pain, and that sedentary time decreased with more pain. Previous studies showed either that PA was inversely related to pain [29, 30] or no relation between PA and pain in children with JIA [23, 24]. JIA often alters the perception of pain and causes decreased pain threshold [31]. An explanation for our results might be that children with JIA that are more active experience more pain similar to every other child, like muscle soreness or pain after detraining. In our study no distinctions was made in pain related to JIA or pain due to PA.

We found that children who feel better (well-being score), appear to move more. This association of PA and well-being has been found previously [4].

Higher functional ability (CHAQ score) was related to less time spend in MVPA, while others did not find this relationship [4, 24, 25]. Children with a higher CHAQ score decrease their MVPA, and had less normal activities in daily life. The PAL was mainly dependent on the low to moderate intensity activities, and not so much on MVPA. This might explain that a relation between CHAQ and MVPA was found, but not with PAL [32].

Despite the fact that this study had a larger sample size and data was collected for a week instead of 1 day or 3 days as in other studies [3, 4, 24, 25], there are some limitations. Data collection of children with JIA was over a longer period of time, so data was collected during the summer and winter periods. As for the controls, the collection only occurred during the summer. PA results differed within the group of children with JIA in favour of the summer. Seasonal variation in physical behaviour in children and adolescents has been found previously [33]. Other studies on PA in children with JIA did not report the season of the data collection [3, 4, 23–25]. Our study was not designed to study the effects of seasonality on PA. Future studies should consider these effects (longitudinal study), or perform measurements in one season.

Another limitation in this study is that data of the control group was collected 2 years earlier than the data collection of the children with JIA. This difference in time might already have resulted in a significant reduction in time spend in MVPA since children tend to become less active over the years. However the percentages of children meeting PA recommendations have been stable over the last few years [27]. No socio-economic variables were available of the controls so we were not able to study effects of these variables, though in the Netherlands healthcare is accessible for all children and all children have equal access to extracurricular sporting activities.

Although an activity diary gives a close estimate of the PAL it still has its limitations [34]. The diary is not an objective instrument and children may under- or overestimate their PA [35]. Studies comparing tri-axial activity monitors with diaries are very needed in this respect.

There might have been selection bias in the participants with JIA. The data of children with JIA came from a larger study of children with JIA willing to participate in a study aimed to improve PA. It is unclear which direction this bias leads since it might be that children willing to participate were less physically active and joined the program for improving PA. On the other hand the group might also consist of children who like to participate in PA and therefore were willing to participate in this program. The girls: boys in this study (2:1) differs from the general JIA population (5:1). It might be that boys are more inclined to sign up for the Rheumates@work program. This difference limits the external validity.

Additionally there were only a few children with disease activity higher than 0.2 cm on the physicians’ global assessment of disease activity since a high disease activity score was an exclusion criteria for participating in the Rheumates@work study. Hence a low PA in children with JIA in this study could not be explained by a high disease activity.

The last limitation in this study is the use of the term sedentary. The term is sometimes used as the lack of exercise. Some studies only describe sitting activities. In this study sitting and lying activities were defined as sedentary time and no distinction was made on lying and sleeping activities in the diary. So it was not possible to make a distinction in sedentary time during the day, which would have given better insights into sedentary activity in children with JIA and controls.

## Conclusions

Although medical treatment of JIA has improved over the years, children with JIA still have a poorer PA compared to controls. Despite encouraging PA in most medical care settings and the growing attention of the importance of PA for pleasure and health benefits, this has not led to an equal amount of PA in children with JIA and controls. Children with JIA need extra help in achieving more normal PA.

## Abbreviations

BMI, body mass index; CHAQ, Childhood Health Assessment Questionnaire; FAO/WHO/UNU, food and agriculture organization of the united nations/world health organization/united nations university; JADAS, Juvenile arthritis disease activity score; JIA, Juvenile idiopathic arthritis; MVPA, moderate to vigorous physical activity; PA, physical activity; PAL, physical activity level; VAS, visual analogue scale

## Appendix 1

Predicted BMR for:

- Boys: 0,074 × body weight (kg) + 2754 MJ/day
- Girls: 0,056 × body weight (kg) + 2898 MJ/day.

To determine the total energy expenditure (TEE), all 15 min periods of each category were summed. Then divided by 96 (total of 15 min periods in a day) and multiplied by the physical activity ratio of each activity category and the predicted BMR.

PAL = TEE/BMR

BMR: basic metabolic rate; kg: kilogram; MJ: mega joule; TEE: total energy expenditure; PAL: physical activity level.
